# Colouration and Colour Changes of the Fiddler Crab, *Uca capricornis*: A Descriptive Study

**DOI:** 10.1371/journal.pone.0001629

**Published:** 2008-02-20

**Authors:** Tanya Detto, Jan M. Hemmi, Patricia R. Y. Backwell

**Affiliations:** 1 Centre for Visual Sciences, Research School of Biological Sciences, Australian National University, Canberra, Australian Capital Territory, Australia; 2 School of Botany and Zoology, Australian National University, Canberra, Australian Capital Territory, Australia; 3 ARC Centre for Excellence in Vision Science, Research School of Biological Sciences, Australian National University, Canberra, Australian Capital Territory, Australia; University of Bristol, United Kingdom

## Abstract

Colour changes in animals may be triggered by a variety of social and environmental factors and may occur over a matter of seconds or months. Crustaceans, like fiddler crabs (genus *Uca*), are particularly adept at changing their colour and have been the focus of numerous studies. However, few of these studies have attempted to quantitatively describe the individual variation in colour and pattern or their adaptive significance. This paper quantitatively describes the colour patterns of the fiddler crab *Uca capricornis* and their ability to change on a socially significant timescale. The most dramatic changes in colour pattern are associated with moulting. These ontogenetic changes result in a general reduction of the colour pattern with increasing size, although females are more colourful and variable than similarly-sized males. *Uca capricornis* are also capable of rapid colour changes in response to stress, but show no endogenous rhythms associated with the semilunar and tidal cycles commonly reported in other fiddler crabs. The extreme colour polymorphism and the relative stability of the colour patterns in *Uca capricornis* are consistent with their use in visually mediated mate recognition.

## Introduction

Many animals are capable of altering their colouration. These changes may take place over a matter of minutes or seconds, and are often involved in social interactions [1], [2], background matching [3], or thermoregulation [4]. Colour changes can also occur on a longer time scale; in response to seasonal changes in the environment [5] or in association with displays during the breeding season [6]. Ontogenetic colour changes occur in association with development, independent of the environment, and are most commonly a response to different anti-predator requirements resulting from changes in life history [7]. The social significance of ontogenetic colour changes has received relatively little attention compared to the more dramatic short-term colour changes.

Ontogenetic colour changes are common in many crustaceans, typically in response to the changing camouflage requirements associated with changes in habitat or behaviour. Upon sexual maturation, female prawns (*Leander serratus*) develop white spots over their egg-bearing segments, possibly because the more vulnerable gravid females require better camouflage [8]. As isopods (*Idotea montereyensis*) grow they change from red to green to match the background as they move from rockpools filled with red algae to beds of green eelgrass [9]. Similarly, shore crabs (*Carcinus maenus*) exhibit white spots on their carapace when small, enabling them to blend into the mussel beds in which they live. As the crabs grow and move into algae beds, they lose their spots to better camouflage themselves [10]. In addition to the variation in colour pattern between juveniles and adults, individual crabs are also extremely variable [11].

Although responsible for some intraspecific variation in colouration, colour changes cannot explain the extreme phenotypic diversity in colour patterns found in many species. While the adaptive significance of short- or long-term colour changes is generally well known, the phenotypic diversity in colour pattern between individuals is often neglected. Whether the colour patterns are individually distinct or sexually dimorphic, as with ontogenetic colour changes, their adaptive significance is often interpreted in terms of predator avoidance [12]–[15]. However, there are a number of benefits associated with being individually recognisable [16], [17]. To fully understand the development and function of colour pattern variation, it is necessary to first describe its morphological basis and the naturally occurring diversity [18].

Fiddler crabs (genus *Uca*) are often spectacularly coloured [19], [20] and capable of dramatic colour changes. Their colour changes have been the focus of numerous studies, resulting in a description of rapid changes associated with courtship displays [21]–[23] and stress [19], [24], [25]. Some fiddler crabs also change colour as a thermoregulatory response to temperature changes [4], [26] and to match the background [4], [20], [27]. Finally, at least 15 species have been found to exhibit rhythmic colour changes in association with their circadian rhythms, and the tidal and semilunar cycles [20], [28], [29]. Despite our detailed knowledge of the physiological processes behind these rhythmic colour changes, their adaptive significance remains a mystery. Furthermore, unlike many other crustaceans, fiddler crabs' ontogenetic colour changes have never been described. *Uca mjoebergi* do not appear to alter their colour and use the colour of their claws to recognise conspecific mates [30]. Species recognition requires a signal that is relatively uniform and stable across the population, whereas recognition of kin, neighbours or individuals requires distinct signals on an increasingly fine scale [31]. While numerous fiddler crab species exhibit extreme individual variations in colour and pattern [19], [24], [32], few studies have attempted to quantitatively describe this diversity [32] and none have examined their adaptive significance.

The endemic Australian fiddler crab *Uca capricornis* Crane, 1975 uses carapace colouration to discriminate neighbours from strangers [30]. Both sexes display a variety of colours on their black carapace, including: blue, yellow, orange and white. In order to function as cues of individual identity, these colour patterns should be relatively stable and include sufficient variation to reliably identify an individual. The aim of this paper is to quantitatively describe the carapace colour patterns of *U. capricornis*, including variation related to sex and size and short- and long-term colour changes.

## Results

### Quantifying Colour Patterns

**Figure 1:**
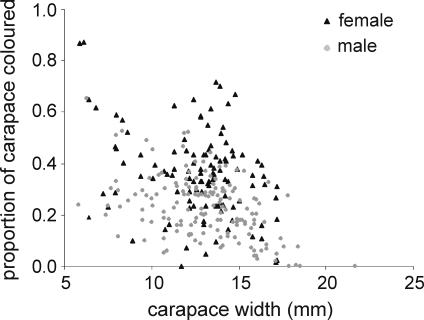
Correlation of colour area and carapace size in male and female *Uca capricornis*. (a) Females are more coloured than males of a similar size, but both sexes become less coloured as they grow. Females n = 115, males n = 158.

**Figure 2:**
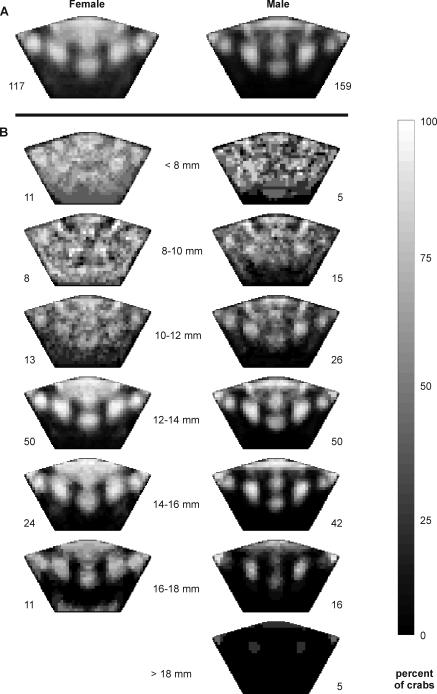
The likelihood that any point on the carapace is coloured. The proportion of males and females in general (a) and within various size classes (b) that are coloured in a particular area is indicated by brightness. Black represents no crabs with colour in that area and white signifies all crabs coloured in that area. Sample sizes are indicated at the base of each image.

Females have a greater proportion of their carapace coloured than a similarly-sized male. The proportion decreases with a corresponding increase in the size of both males and females (GLM arcsine proportion carapace coloured: carapace width F_1, 272_ = 76.6, p<0.0001; sex F_1, 272_ = 50.9, p<0.0001; interaction F_1, 272_ = 0.113, p = 0.7) (Figure 1 and 2). The result is that juveniles of both sexes have uniformly coloured carapaces, but the coloured area contracts in larger individuals, giving them a mottled pattern, before concentrating into 6 particular areas (Figure 2). Crabs larger than approximately 12 mm generally exhibit some form of this pattern, consisting of a patch behind the eyestalks (the scarf), surrounded by another 5 spots. The exact size of these patches varies between individual crabs; from non-existent, to covering much of the carapace.

**Figure 3:**
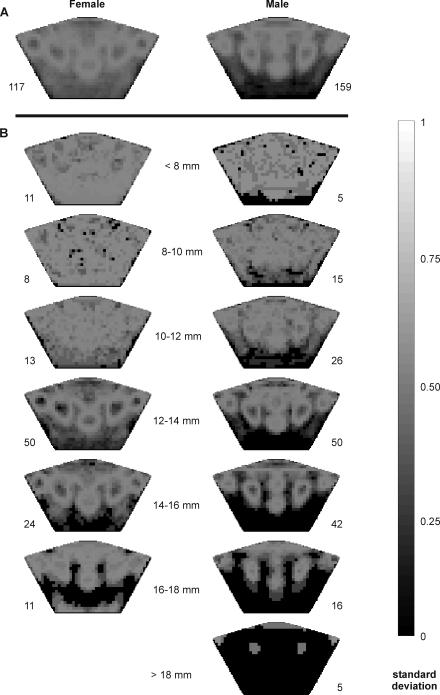
Variation in the probability that any particular area of the carapace is coloured. The standard deviation in the number of males and females in general (a) and in different size classes (b) that are coloured at each point on the carapace. Lighter greys indicate greater variation. The sample sizes are indicated at the base of each image.

Juvenile crabs have the most variable colour patterns, due to their mottling (Figure 3b). Individuals larger than 12 mm vary in the size of the 6 specific areas (Figure 3b), with females' spots tending to be larger and more variable (8.3±10.5 mm^2^, n = 82 ) than males' (2.7±3.3 mm^2^, n = 111) (Figure 3a).

### Rapid Changes

**Figure 4:**
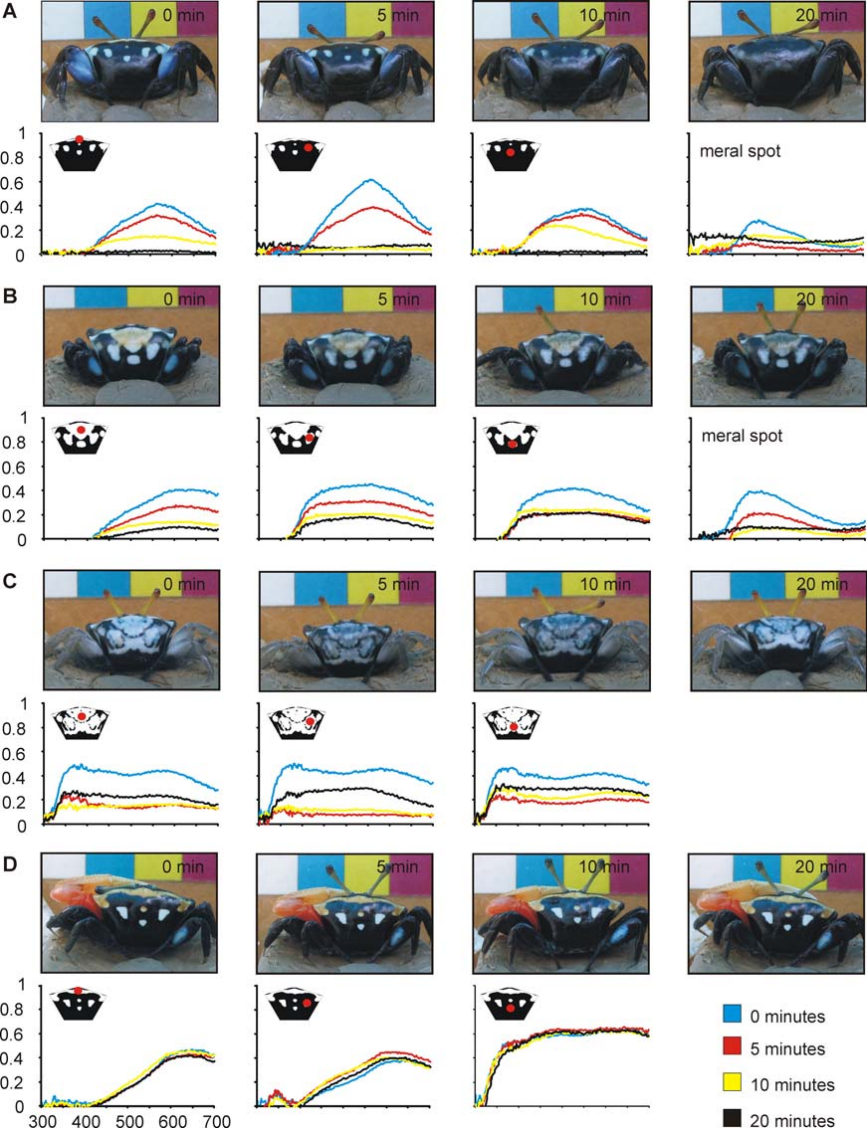
Rapid colour changes in *Uca capricornis* in response to capture. Each graph corresponds to the spectral reflectance of the area indicated in red on the stylised carapace. Measurements were taken at 0 (blue), 5 (red), 10 (yellow) and 20 (black) minutes after capture. Most individuals steadily darkened their entire colour pattern (a), while in others the yellow scarf region and blue meral spots were more likely to darken (b). Some began to regain their colour within the 20 minutes (c). A small number of crabs, particularly males, did not change colour over 20 minutes (d).

When caught, or otherwise stressed, *U. capricornis* are capable of rapidly changing their colour. These short-term colour changes involved darkening their colour to some degree, based on human observation, and were more common in females (35 out of 43) than males (12 out of 22) (2-tailed Fisher's exact test, p = 0.04). Eighteen crabs darkened their entire colour pattern equally (Figure 4a). In another 17 crabs, the yellow scarf region darkened more noticeably than the white spots (Figure 4b). While these crabs became steadily darker over the 20 minutes, the remaining 12 crabs darkened as normal but started to brighten, and in some cases almost regained their original colouration, within the 20 minutes (Figure 4c). In all cases the pattern remained unchanged, but did become more difficult to distinguish as the colours darkened.

### Daily Changes

*Uca capricornis* do not regularly change their colouration over the course of a day. Of the 14 females and 13 males observed throughout a day, the vast majority (13 and 11 respectively) did not noticeably change their colour, and none altered their pattern.

**Figure 5:**
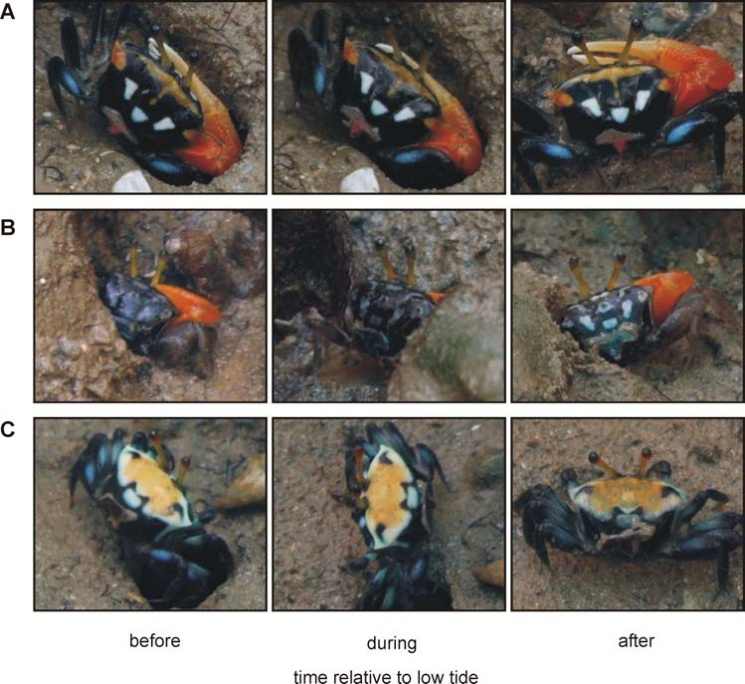
Colour changes in *Uca capricornis* before, during and after low tide. One male brightened primarily in the yellow scarf region (a), while the other brightened all over (b). The only female that changed colour darkened in response to an encounter with another crab after low tide (c).

The two males that did change brightened their colour slightly over the course of the day, peaking after low tide. In the larger male the change was most pronounced in the yellow scarf region (Figure 5a), while the smaller black and white male brightened all over (Figure 5b). On the other hand, the single female that did change darkened after low tide (Figure 5c), apparently in response to being evicted from her burrow by another crab. These changes appeared quite subtle compared to the rapid changes described above.

### Changes with the Semilunar Cycle

**Figure 6:**
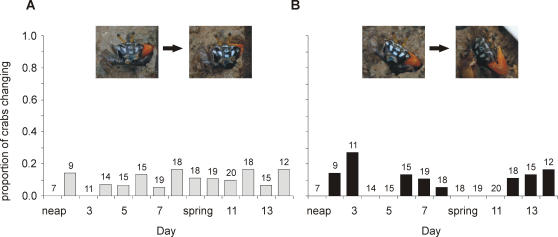
Colour change in *Uca capricornis* in relation to the semilunar cycle. The proportion of crabs that brightened (a), or darkened (b) on each day of the tidal cycle relative to the number of crabs active on that day, which is presented above each bar. Examples of a crabs brightening and darkening are presented in the appropriate graphs.

*Uca capricornis* do not rhythmically change their colour or pattern between moults in association with the semilunar cycle. No crabs noticeably changed their pattern or from one colour to another, and 12 out of 22 did not alter their colour in any way from one day to the next over a two week cycle. The remaining 11 crabs brightened or darkened their existing colours slightly from one day to the next. However, the number of changes made by each crab over the 14 day cycle varied, from 1 change (3 crabs), 2 changes (2 crabs), 4 changes (1 crab), 5 changes (3 crabs), 6 changes (1 crab), or 7 changes (1 crab). There was no apparent trend in the number of crabs either brightening or darkening with respect to the time in the cycle; there was a low level of both brightening and darkening, throughout the 2 weeks (Figure 6).

### Changes Associated with Moulting

**Figure 7:**
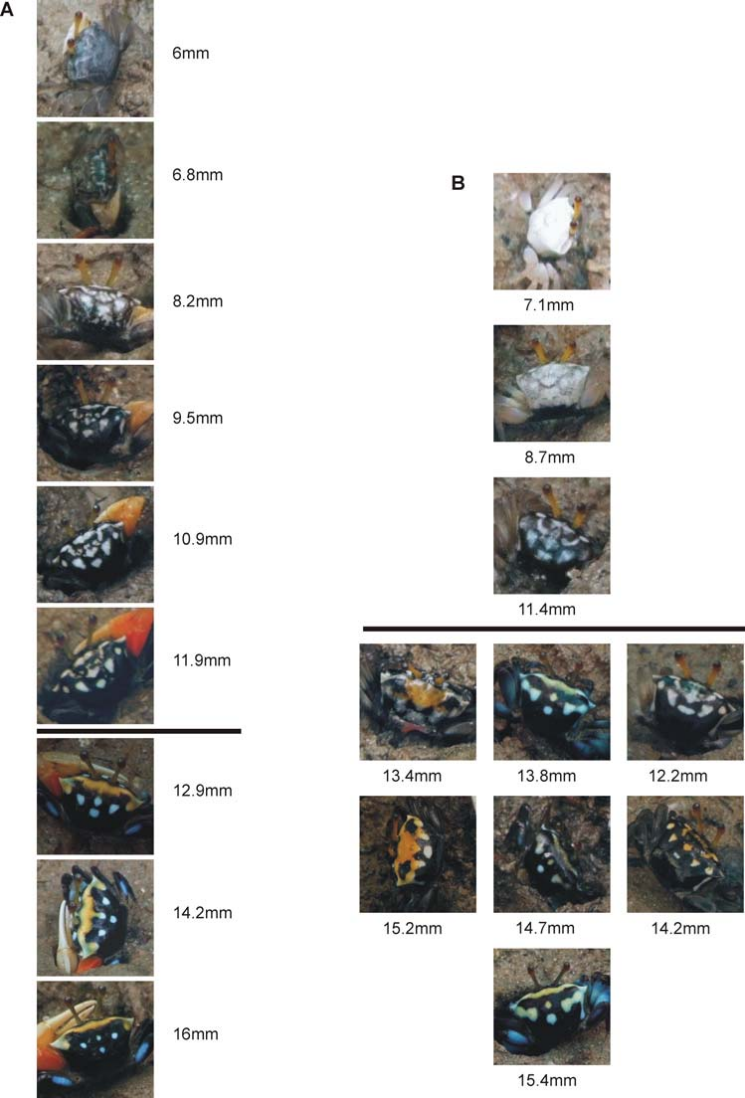
Changes in colouration associated with moulting in *Uca capricornis*. (a) The typical male progression from a mottled black and white pattern, to distinct white spots with a yellow scarf is illustrated by the successive moults of 2 males, separated by a line. (b) Females have a more variable progression. The successive moults of one small individual are presented to represent the less colourful phase, and three individuals are presented to illustrate the more variable colour patterns of larger females, with their corresponding carapace widths below.

On average, *U. capricornis* grew 1.6 mm with every moult (standard deviation = 0.5 mm, n = 37). The difference in pattern between individuals of different sizes is clearly the result of changes within individuals associated with moulting, as the correlation between colour pattern and size is consistent with the changes observed in moulting individuals. With each successive moult the pattern appears to contract and become more distinct, especially in males (Figure 7).

In addition to the considerable changes in pattern, both sexes also changed the specific colour of their spots. The colour changes associated with moulting, as with changes in pattern, tend to follow a certain progression (Figure 7). The first few moults of both sexes are a uniformly brilliant light blue, which pales to white over successive moults. They retain this black and white colouration over the next 3–5 moults, as they develop the mottled pattern and the beginnings of the distinctive scarf and spots. Upon reaching about 12 mm males and females start to differ.

In males the scarf and the two spots at the corners of their carapace turn yellow, while the lower spots generally remain white (Figure 7a). In addition to the yellow on their carapace, males over 12 mm also develop distinctive blue meral spots on their fourth pair of walking legs, which are retained by the largest individuals that have otherwise lost all carapace colour. Females larger than about 12 mm also develop more colourful carapaces, although they are more variable than those of males: their scarf and surrounding spots can be any combination of yellow, white, or blue (Figure 7b). As with males, larger females can also develop blue meral spots however, these are not restricted to the last pair of legs, and may cover the entire leg.

After developing a yellow scarf, males do not change the colour of their patches again. The coloured areas steadily shrink over successive moults, until they disappear entirely (Figure 7a). On the other hand, although the colour and pattern of the 6 caged females large enough to develop colour remained relatively stable over successive moults, when they did change they were more variable and less likely to follow a certain progression (Figure 7b).

### Perception of Colour Patterns

**Figure 8:**
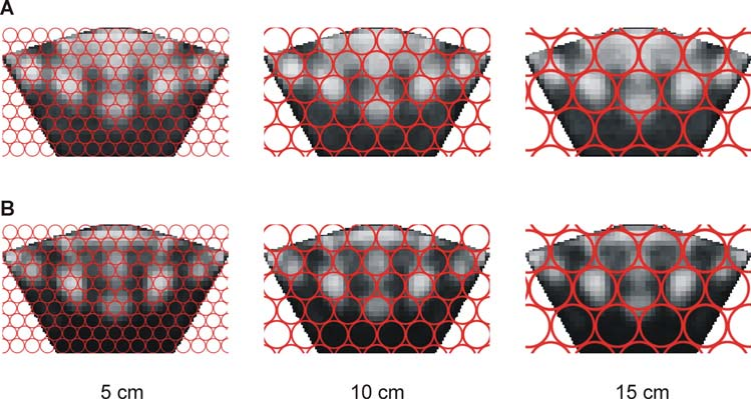
The theoretical perception of average colour patterns by *Uca capricornis*. The dorsal view of the carapace has been projected onto the ommatidial array (indicated by the red circles), for viewing distances of 5, 10 and 15 cm. The various colour patches of both females (a) and males (b) are theoretically distinguishable at 5, 10, and 15 cm.

Based on the theoretical visual acuity of *U. capricornis*, the spacing between the receptive fields of ommatidia would be 0.9 mm for a viewing distance of 50 mm, 1.7 mm for 100 mm, and 2.6 mm for150 mm. The configuration of their typical colour patches appears to enable them to be detected by individual, non-adjacent ommatidia from as far away as 15 cm (Figure 8). This should enable *U. capricornis* to perceive the variation in colour pattern in both females (Figure 8a) and males (Figure 8b).

## Discussion

The colour patterns on the carapaces of *Uca capricornis* are extremely variable, although they do follow a certain progression with size and sex. Both males and females change from a uniform blue colour to a pattern of distinct white, yellow, or blue patches on a black background. The coloured patches of both sexes tend to shrink as the crabs grow, but females are generally more variable, with a wider variety of colours in larger patches than similarly-sized males.

Habitat partitioning and differential predation pressure are linked to sexually dimorphic colour patterns in other crustaceans, such as the isopod *Idotea baltica* [13], [14] and are involved in maintaining colour polymorphism in many species, including vertebrates [12]. There is no apparent habitat partitioning in *U. capricornis* based on sex, size, or colour morph. However, the different colour classes do appear to behave differently as larger individuals are more inclined to leave their burrows and wander through the colony (T Detto & PRY Backwell unpublished data). This behaviour may expose them to a greater risk of predation, which may select for their less colourful appearance. However, this cannot be proven until we identify their predators and determine their visual capabilities.

A likely explanation for the extreme variation in colour patterns in *U. capricornis* is their involvement in individual discrimination. Males live in close association with their neighbouring females (T Detto & PRY Backwell unpublished data) and use carapace colour patterns to distinguish between their female neighbour and unknown females [30]. The more variable colour patterns of females appear to be unique and may enable their mates to distinguish them from other females. Models of the theoretical perception of the average colour pattern suggest that the crabs are physiologically capable of discriminating variation in spot size and distribution. Recent work has also shown that another species, *U. mjoebergi*, possess colour vision [33], which may also mean *U. capricornis* can make use of the individual variation in colour.

In addition to the ability to perceive variation in colour pattern, an individual's colour pattern needs to be relatively stable over time to function as an individual identifier, as colour changes may result in misidentification. A number of fiddler crabs reportedly change their colour around spring tide and over low tide, when waving and courtship activity is at a maximum [19], [22], [23], [25]. We found no evidence that *U. capricornis* regularly change their colour in association with the semilunar or tidal cycle. The few crabs that did change their colour during the semilunar and tidal cycle were likely doing so in response to an unobserved social stimulus rather than an endogenous rhythm. *Uca capricornis* are capable of rapidly changing their colour in response to capture or losing their burrow. This colour change involves darkening existing colours, probably by temporarily concentrating the coloured pigments and/or dispersing black pigment. The pattern remains unchanged and all evidence suggests that the crabs regain their original colouration. It is difficult to say what adaptive significance these rapid colour changes have. They may be used in intraspecific communication, as a form of camouflage for crabs that have lost their burrow, or they may simply be a physiological response to stress.

While *U. capricornis* are clearly capable of rapid physiological colour changes, these do not appear to play a major role in the crabs' general colouration. Their colouration is determined by ontogenetic colour changes during moulting. Between moults, their colour pattern is very stable. They seem unable to change the position, number, or colour of their chromatophores between moults. While they are able to dull their colour when stressed, they then return to their original state. The carapace colour patterns of *U. capricornis* are thus stable enough to allow individual discrimination: they remain the same during an entire inter-moult period, which is likely longer than pairs remain together (T Detto & PRY Backwell unpublished data). Furthermore, larger females maintain a relatively similar colour pattern over several moults.

In addition to individual identity, the ontogenetic colour changes in *U. capricornis* potentially provide information about the sex, size, and reproductive status of the individual. The crabs do not develop their yellow colouration until growing to about 12 mm. This may be because they need time to accumulate the necessary carotenoids, or it may be a signal of sexual maturity. Female prawns and shrimps develop white chromatophores at sexual maturity [8]. The steadily decreasing size of the coloured patches in larger males also has the potential to signal competitive ability.

When first described, *Uca capricornis* was believed to be 2 distinct species: *U. capricornis* and *U. pavo*, based on the extreme difference in colouration between males of different sizes [34]. Together with the potential social significance of colour in *U. capricornis*, this highlights the importance of studying ontogenetic colour changes and our lack of understanding of their possible significance.

## Materials and Methods

### General Methods

This study was conducted on a large population of *Uca capricornis* in the vicinity of the mangrove boardwalk in the East Point Reserve, Darwin, Australia (12°24′35″ S, 130°50′00″ E). Fieldwork was conducted from November to January 2004/2005 and 2005/2006. References to low tide are based on the predictions of the National Tidal Facility (Flinders University) for Darwin harbour.

Several experiments were conducted on crabs housed within cages built on the mudflat, these cages are discussed in detail in the final section (changes associated with moulting).

Every effort was taken to minimise any possible effect of our presence on the behaviour of the crabs, including those in the cages, as they undergo a dramatic colour change when directly handled. Experience has proven *Uca capricornis* to be particularly resilient to human presence, and within 5 minutes of motionless observation the crabs resumed an apparently normal range of behaviour, often next to or even on top of the observer's feet. From this we have to assume that our presence had little impact on the behaviours reported below. Unfortunately, this also required us to rely on the human visual system to describe many of the long-term colour changes

### Quantifying Colour Patterns

Colour pattern refers to the configuration of any coloured areas on the black carapace, regardless of their spectral composition. To quantify the carapace colour patterns of *U. capricornis* we caught and measured the carapace width of all individuals active on the surface within 18 plots (4 m^2^ each) and a random sample of larger crabs, which were relatively sparse within the plots. We thus collected a total of 117 females and 159 males. We temporarily restrained them before taking a digital photograph of their carapace. Afterwards, the crabs were released unharmed.

All digital photographs were taken using manual white balance and included a colour standard. We used the manual colour correction function of Paint Shop Pro version 7.02 (Jasc Software 2000) to standardise the colour of the photographs and account for lighting differences. This allowed more accurate comparisons of individual differences and colour changes within individuals.

Using a raster-based graphics software [Paint Shop Pro version 7.02 (Jasc Software 2000)] we converted each crab carapace into a stylised black hexagon, with corners at the base of the last pair of legs, the widest points of the carapace, and the base of the eyestalks. We then converted any colour markings on the carapace to white before standardising the size and orientation of the images using ENVI software (Research Systems Colorado) to warp them to a standard carapace 45 mm wide and 25 mm high. The resulting images showed the relative position of the colour pattern (in white) on a black carapace.

We quantitatively examined the relationship between colouration, carapace size, and sex. Using the public domain Scion Image (Alpha 4.0.3.2) program (Scion Corporation), we measured the total size of the coloured area and the area of the carapace, using the measured carapace width as a scale. We then determined the proportion of the carapace that was coloured and examined its relationship with carapace size and sex using a generalised linear model. The data were tested for normality, and the proportion of the carapace that was coloured was arcsine transformed to conform to the assumptions of normality. Furthermore, we calculated the average size of the various colour patches by dividing the total coloured area by the number of distinct spots. Means are reported with the standard deviations.

To visualise the differences in colour pattern between the sexes and crabs of different sizes, we constructed images illustrating the probable colour patterns of various groups of crabs, including: males and females in general, and males and females within various size classes; <8 mm, 8–9.9 mm, 10–11.9 mm; 12–13.9 mm; 14–15.9 mm; 16–17.9 mm; and males >18 mm (no photographed females were >18 mm). We overlayed a 1×1 mm grid on each of the warped images and assigned a value of 1 to each coordinate that was more than one third white and 0 to all other, uncoloured, coordinates. We then determined the proportion of the crabs that had colour at each coordinate and converted these percentages into RGB lightness values, with 0 (black) equivalent to no crabs coloured in that area and 255 (white) equivalent to all crabs coloured in that area. We used these values to colour the appropriate coordinates on a standardised carapace, producing an image that represents the likelihood that any particular area is coloured. These images are the equivalent of an average crab's colour pattern. To more clearly illustrate the variation in colour pattern between the groups, we constructed similar images using the standard deviation of crabs coloured at each coordinate.

### Rapid Changes

We examined the ability of 65 randomly caught crabs (21 males and 44 females) to change colour over 20 minutes. We measured the spectral reflectance of various coloured areas on their carapace relative to a white ‘Spectralon’ standard with a USB2000 UV-VIS portable spectrophotometer (Ocean Optics Inc, Dunedin, FL, USA). The first measurements were taken as soon as possible after capture, and further measurements were taken of the same areas 5, 10 and 20 minutes after the initial measurements. After each set of measurements we also took a digital photograph. Between measurements, the crabs were kept in the shade in a clear plastic cup with 1 cm of seawater.

### Daily Changes

We also examined whether *U. capricornis* change colour in relation to the tidal cycle. On the day after spring tide, over several semilunar cycles, we observed 27 crabs (13 males and 14 females), including those within the cages and several outside. We took digital photographs of each crab 2 hours to 1 hour before low tide (before), in the 1 hour around low tide (during), and 1 hour to 2 hours after low tide (after). Each crab thus had three photographs taken over a single day, each separated by 1.5 hours. We then examined the photographs be eye for any changes in colour or pattern based.

### Changes with the Semilunar Cycle

During one semilunar cycle, from one neap tide to the next, we took digital photographs of all 22 crabs (10 males and 12 females) active in the cages at approximately low tide every day. We examined the photographs and identified the proportion of the crabs that were darker, brighter, or the same colour as the previous day to determine whether they underwent any rhythmic colour changes. As we only observed them over a single cycle we cannot make any definitive statements about their colour changes associated with the semilunar cycle, but can identify any obvious trends.

### Changes Associated with Moulting

We constructed 5 circular cages (1 m in diameter) out of flyscreen mesh and buried them 10 cm in the mud within the *U. capricornis* population. The cages protruded a further 20 cm above the mud and a laminate strip glued around the inner and outer top 5 cm prevented crabs from climbing into or out of the cage. We removed all existing crabs from the cages and restocked each cage with 4 crabs; a small male and female and a large male and female. We measured the crabs, took a digital photograph of their carapace and superglued a coloured sequin to the posterior region of their carapace. Checking on the crabs every week, we identified and photographed those lacking a sequin, indicating they had recently moulted, before measuring and remarking them. Over the 4 months of the experiment we had to replace several of the crabs that disappeared, and although several reappeared, they were distinguishable by their size, colour pattern and the colour of their sequin. In total we observed and photographed 11 females and 10 males through at least one moult.

### Perception of Colour Patterns

Finally we examined whether *Uca capricornis* are anatomically capable of perceiving the different colour patterns, by using a theoretical inter-ommatidial angle of 1° [35], [36] as an estimate of the crabs' maximum spatial resolution. We calculated the spacing between the centres of adjacent ommatidia for distances of 50, 100, and 150 mm.
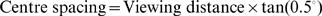



We then assigned the standardized image of an average carapace (calculated while quantifying the colour pattern) a width of 14 mm, and projected this image on the crab's ommatidial array (Figure 8).The crabs should be able to discriminate the various colour patches if they are perceived by individual, non-adjacent ommatidia.
